# Designing a model of professional ethics excellence for clinical librarians

**DOI:** 10.5195/jmla.2020.893

**Published:** 2020-10-01

**Authors:** Hasan Ashrafi-rizi, Zahra Kazempour, Fatemeh Sheikhshoaei, Zahra Ghazavi

**Affiliations:** 1 hassanashrafi@mng.mui.ac.ir, Professor, Health Information Technology Research Center, Isfahan University of Medical Sciences, Isfahan, Iran; 2 zahrakazempour00@gmail.com, Assistant Professor, Library and Information Science, Department of Knowledge and Information Science, Payame Noor University, Tehran, Iran; 3 (corresponding author), fashoaei@sina.tums.ac.ir, Assistant Professor, Medical Library and Information Sciences, School of Allied Medicine, Tehran University of Medical Sciences, Tehran, Iran; 4 ghazaviz@ymail.com, Iran University of Medical Sciences, Tehran, Iran

## Abstract

**Objective:**

Developing and promoting professional ethics principles for clinical librarians can help the health care system balance the interests of all stakeholders, including clinical librarians, health care professionals, and patients. Therefore, the goal of this study was to design a model of professional ethics excellence for clinical librarians.

**Methods:**

The authors conducted a descriptive applied study using literature review and the delphi method. The delphi panel included eleven experts in medical librarianship, library and information sciences, or information sciences and knowledge studies.

**Results:**

After the delphi rounds, five concepts and forty-six components were identified and confirmed to provide a model of professional ethics excellence for clinical librarians. The highest-rated concept was excellence in communication. The highest-rated component was mastery in developing search strategies in information resources and databases.

**Conclusions:**

Identifying and applying principles of professional ethics among clinical librarians can enhance the professionalization of clinical librarians and result in better information services for physicians. Furthermore, incorporating these principles into the curriculum for health sciences library and information sciences students or into workshops for active clinical librarians can further formalize the profession and practice of evidence-based medicine.

## INTRODUCTION

Professional ethics encompasses a set of ethical principles to which practitioners in a profession agree to adhere and forms an important part of one's professional life [1]. This is especially true for health care providers and medical information–related professionals such as librarians, informationists, and medical information technology professionals, because the disclosure of patient-related information or failure to provide timely and accurate information to health care providers and patients can inflict irreparable damage to the treatment process. The use of valid and up-to-date information in the prevention, diagnosis, treatment, and rehabilitation processes can save lives, reduce medical costs, and improve the quality of health services [2].

In the nineteenth century, Mary W. Plummer, head of the School of Library and Information Science at the Pratt Institute, laid down professional ethics principles and regulations for library practitioners such as physicians, lawyers, clergymen, and university professors. However, professional ethics rules were not formally adopted until the American Library Association (ALA) published the Library Bill of Rights in 1939 and Freedom to Read Statement in 1953 [3, 4]. Since then, international organizations such as the International Federation of Library Associations and Institutions (IFLA) [5] and the Medical Library Association (MLA) [6] have developed additional professional codes of ethics for health sciences librarians.

However, there has been no comprehensive attempt to compile professional ethics codes for clinical librarians, who should have their own principles due to their particularly close ties to the health care sector and potential influence on patient care. Clinical librarians are present in treatment teams and wards to help health care professionals search for and evaluate the best evidence and to promote evidence-based medicine (EBM) principles [7]. Finding the best evidence is a valuable skill that clinical librarians possess, because physicians may have difficultly sifting through the large amount of available medical information and distinguishing between credible and unreliable sources. Indeed, clinical librarians have mastery over the design and execution of database search strategies, critical appraisal skills, knowledge of medical terminology, and good communication with health care professionals [7–11].

Clinical librarians who engage in the medical information–seeking process alongside health care professionals must adhere to principles of professional ethics to foster their professional growth and excellence in clinical settings and enhance the quality of care provided to patients. Learning and adhering to professional ethics standards can boost the effectiveness of well-trained and experienced clinical librarians in providing the best evidence for health care professionals to use in making clinical decisions [12]. A review of the literature shows that research on professional ethics in librarianship has mainly examined the practice of professional ethics principles among librarians in general or medical librarians [13–21] or has reformulated these principles for use in different countries and fields [3, 5, 6, 19, 22–24]. In addition, some research points to a slight difference between professional ethics for librarians in general and medical librarians in particular [3, 20].

What remains to be addressed, however, is the development and promotion of professional ethics principles for clinical librarians, which could aid in the training of new clinical librarians, enable the evaluation and comparison of professional ethics practices at and among different health care centers, and allow the health care system to better balance the interests of all stakeholders (i.e., clinical librarians, health care professionals, patients). Therefore, the goal of this study was to design a model of professional ethics excellence for clinical librarians.

## METHODS

The authors performed an applied descriptive study via literature review and the delphi method. The delphi method is a process of obtaining expert group opinions on a topic [25, 26] that seeks to align different experts' viewpoints by systematically refining their responses [27]. We selected 11 panel members based on non-probability purposive sampling. Panel members (men, n=7; women, n=4) consisted of faculty members in medical librarianship (n=4), library and information sciences (n=1), or information sciences and knowledge studies (n=6) at 8 different universities (Ahvaz University of Medical Sciences, Allameh Tabataba'i University, Bushehr University of Medical Sciences, Iran University of Medical Sciences, Iranian Research Institute for Information Science & Technology, Isfahan University of Medical Sciences, Kerman University of Medical Sciences, and Shahid Beheshti University of Medical Sciences) who had a background in education or research in clinical librarianship or professional ethics in the field of medical librarianship. The highest degree of 9 panel members was a doctorate (PhD), whereas 2 had a master's degree. Two panel members were associate professors, 7 were assistant professors, and 2 were lecturers.

For the first delphi round, we prepared a questionnaire identifying 5 concepts and 48 components based on relevant scientific literature [3, 5–7, 10, 11, 13, 20, 28–32] and our own experiences (supplemental Appendix A). To increase the participation of panel members, the purpose of the questionnaire, potential utility of the results, affected stakeholders, panel member confidentiality, and number of possible delphi rounds were described at the beginning of the questionnaire. Questionnaire items utilized a 10-point Likert scale, on which panel members rated the importance of components from low importance (response of “1”) to very important (response of “10”). In addition to the concepts and components provided, we also asked panel members to propose additional concepts or components.

Obtaining panel member responses in the first round took approximately 1 month. Three components had a mean rating less than or equal to 7, which we considered to indicate a lack of panel member consensus; however, these components were retained in the second-round questionnaire. Also, 1 new concept and 3 new components were proposed by the panel members and were included in the second-round questionnaire, so we revised the descriptions of some components.

For the second delphi round, a new questionnaire identifying six concepts and fifty-one components was sent to the panel members (supplemental Appendix B). We described the changes made to the components based on the first-round results as well as the mean concept and component ratings in the first round at the beginning of the questionnaire. Ten of the eleven panel members participated in the second round, which took approximately two weeks. The three components that had a mean rating less than or equal to seven in the first round did not receive a mean rating greater than seven in the second round and, thus, were omitted. Also, one concept and two components proposed by the panel members in the first round did not receive a mean rating greater than seven in the second round and, thus, were omitted. Therefore, after two rounds of delphi, five concepts and forty-six components were included in the final model.

## RESULTS

In the first delphi round, the highest-rated concept was excellence in performance, and the lowest-rated concept was excellence in education (Table 1). The highest-rated component was the role and influence of clinical information services on the level of care received by patients, and the lowest-rated component was clinical librarian availability even during non-office hours. At the end of the first round, the panel members reached consensus on 5 concepts and 45 components (i.e., mean rating >7).

**Table 1:** First round of delphi panel member ratings

Concept	Component		Mean rating
Education excellence (mean of 7.84)	1.	Converts health care professionals into practitioners of evidence-based medicine (EBM)	7.63
	2.	Continues to educate health care professionals about clinical information literacy	8.36
	3.	Continues to educate residents and interns about clinical information literacy	8.12
	4.	Updates one's specialized knowledge and that of other librarians by participating in continuing education	9.09
	5.	Engages in practical in-service training in clinical librarianship	8.54
	6.	Recommends and updates medical librarianship curriculums with a focus on clinical librarianship	6.72
	7.	Trains a new generation of clinical librarians to support EBM	7.00
	8.	Takes advantage of the knowledge and expertise of leaders in clinical librarianship	7.45
Performance excellence (mean of 8.27)	9.	Demonstrates relative command of terms and concepts used by health care professionals	8.27
	10.	Masters search strategies in information resources and databases	
	11.	Masters clinical question formation (e.g., problem, intervention, comparison, outcome [PICO])	8.40
	12.	Demonstrates sufficient knowledge and mastery of evidence-based information sources	8.27
	13.	Pays attention to the clinical information needs of health care professionals	8.27
	14.	Provides reliable and up-to-date information (i.e., best evidence) for health care professionals	8.20
	15.	Considers patient values in the EBM process	7.80
	16.	Quickly and accurately responds to the clinical questions of health care professionals	8.18
	17.	Supports accurate clinical decisions and records experiences	7.63
	18.	Attends clinical rounding	8.00
	19.	Maintains patient privacy	8.63
	20.	Believes in the role and influence of clinical information services on the level of care received by patients	9.18
	21.	Has a timely and effective presence in the clinical setting	8.00
Communication excellence (mean of 7.91)	22.	Appropriately and respectfully communicates with health care professionals and patients	8.36
	23.	Enjoys good self-esteem when interacting with health care professionals	8.36
	24.	Shows confidence in communicating with health care teams	8.45
	25.	Collaborates with health care teams to facilitate the EBM process	9.00
	26.	Utilizes one's scientific ability and talent and that of other librarians to provide appropriate services to health care professionals	7.90
	27.	Actively interacts with and shows respect to other librarians	7.81
	28.	Accepts constructive and wise feedback from other librarians and health care professionals	8.45
	29.	Respects the job performance of other librarians	7.90
	30.	Has insight into the information behavior and performance of health care professionals	7.54
	31.	Avoids inappropriate jokes when performing job activities	7.27
	32.	Has a professional physical appearance in the workplace	7.90
	33.	Effectively communicates with senior executives of the organization to support and enhance EBM and clinical librarianship	8.18
	34.	Gains the necessary communication skills for interacting with others	7.40
	35.	Is available even during non-office hours when necessary	5.60
Research excellence (mean of 8.13)	36.	Supports research related to clinical librarianship and EBM	8.27
	37.	Supports rationale for using best evidence in clinical decision-making	8.63
	38.	Identifies clinical information needs of health care professionals based on scientific research	8.27
	39.	Develops scientific and teaching resources related to clinical librarianship and EBM with an emphasis on new concepts, theories, and local needs	7.18
	40.	Develops clinical librarian programs and services based on valid research findings	8.36
Professional status excellence (mean of 8.14)	41.	Believes in the existential philosophy of clinical librarianship to effectively enhance clinical information services provided to health care professionals	8.63
	42.	Believes in evidence-based clinical librarianship and its formalization	8.45
	43.	Gains the trust of health care professionals in the capabilities of clinical librarians in the context of EBM	7.63
	44.	Strengthens and expands the independent and effective identity of clinical librarians among the general public and health care professionals	8.00
	45.	Believes in professional cohesion and moving toward common interests	7.90
	46.	Strives to maximize the usefulness of clinical information services	8.27
	47.	Strives to promote the status of clinical librarians at national and international levels	8.27
	48.	Upgrades the level of professional integration of clinical librarianship	8.00

In the second delphi round, the highest-rated concept was excellence in communication, and the lowest-rated concept was excellence in performance (Table 2). The highest-rated component was the mastery of search strategies in information resources and databases, and the lowest-rated component was the presence of clinical librarian feedback to influence the treatment process. At the end of the second round, the panel members reached consensus on all five concepts and forty-six components.

**Table 2:** Second round of delphi panel member ratings

Concept	Component		Mean
Education excellence (mean of 8.01)	1.	Converts health care professionals into practitioners of EBM	7.40
	2.	Continues to educate health care professionals about clinical information literacy	8.10
	3.	Continues to educate residents and interns about clinical information literacy	7.90
	4.	Updates one's specialized knowledge and that of other librarians by participating in continued education	9.40
	5.	Engages in practical in-service training in clinical librarianship	7.80
	6.	Takes advantage of the knowledge and expertise of leaders in clinical librarianship	7.50
Performance excellence (mean of 7.98)	7.	Demonstrates relative command of the terms and concepts used by health care professionals	8.80
	8.	Masters search strategies in resources and databases	9.60
	9.	Masters clinical question formation (e.g., PICO)	9.20
	10.	Demonstrates sufficient knowledge and mastery of evidence-based information sources	9.20
	11.	Pays attention to the clinical information needs of health care professionals	8.90
	12.	Provides reliable and up-to-date information (i.e., best evidence) for health care professionals	9.30
	13.	Considers patient values in EBM process	8.33
	14.	Quickly and accurately responds to the clinical questions from health care professionals	9.20
	15.	Supports accurate clinical decisions and records experiences	7.80
	16.	Attends clinical rounding	8.60
	17.	Maintains patient privacy	8.80
	18.	Believes in the role and influence of clinical information services on the level of care the patient receives	9.00
	19.	Has a timely and effective presence in the clinical setting	8.50
	20.	Provides feedback to influence the treatment process	8.80
Communication excellence (mean of 8.55)	21.	Appropriately and respectfully communicates with health care professionals and patients	8.80
	22.	Enjoys good self-esteem when interacting with health care professionals	9.20
	23.	Shows confidence in communicating with health care teams	9.12
	24.	Collaborates with health care teams to facilitate the EBM process	9.00
	25.	Utilizes one's scientific ability and talent and that of other librarians to provide appropriate services to health care professionals	8.30
	26.	Actively interacts with and shows respect to other librarians	8.70
	27.	Accepts constructive and wise feedback from other librarians and health care professionals	8.80
	28.	Respects the job performance of other librarians	8.50
	29.	Has insight into the information behavior and performance of health care professionals	8.11
	30.	Avoids inappropriate jokes when performing job activities	7.60
	31.	Has a professional physical appearance in the workplace	8.60
	32.	Effectively communicates with senior executives of the organization to support and enhance EBM and clinical librarianship	8.50
	33.	Gains the necessary communication skills for interacting with others	8.10
Research excellence (mean of 8.04)	34.	Supports research related to clinical librarianship and EBM	7.90
	35.	Supports rationale for using best evidence in clinical decision-making	8.20
	36.	Identifies clinical information needs of health care professionals based on scientific research	8.55
	37.	Develops scientific and teaching resources related to clinical librarianship and EBM with an emphasis on new concepts, theories, and local needs	7.40
	38.	Develops clinical librarian programs and services based on valid research findings	8.20
Professional status excellence (mean of 8.45)	39.	Believes in the existential philosophy of clinical librarianship to effectively enhance clinical information services provided to health care professionals	8.40
	40.	Believes in evidence-based clinical librarianship and its formalization	8.60
	41.	Gains the trust of health care professionals in the capabilities of clinical librarians in the context of EBM	8.40
	42.	Strengthens and expands the independent and effective identity of clinical librarians among the general public and health care professionals	8.40
	43.	Believes in professional cohesion and moving toward common interests	8.30
	44.	Strives to maximize the usefulness of clinical information services	8.77
	45.	Strives to promote the status of clinical librarians at national and international level	8.50
	46.	Upgrades the level of professional integration of clinical librarianship	8.33

## DISCUSSION

Based on the results obtained in the second delphi round, five concepts and forty-six components were identified and confirmed in order to develop a model of professional ethics excellence for clinical librarians. The highest-rated concept was communication excellence, and the lowest-rated concept was performance excellence. The highest-rated component was mastering search strategies in information resources and databases, and the lowest-rated component was the presence of clinical librarian feedback to influence the treatment process.

### Education excellence

Clinical librarians should update their specialized knowledge and that of their librarian colleagues by attending continuing education courses aimed at helping them improve clinical information literacy among health care professionals and provide context for using reliable information resources and databases in the practice of EBM. Previous studies have addressed these educational issues and emphasize that clinical librarians should effectively teach information literacy concepts in the EBM process [33, 34], because high-quality implementation of EBM in clinical centers depends on the training of both clinical librarians and physicians [10, 35]. Given the speed of production and dissemination of information resources, being up to date is a prerequisite for dynamically providing high-quality health care [20] and requires specific training. Furthermore, it is important that managers of health sciences libraries receive continuing education to update their knowledge about ethical issues [21].

### Performance excellence

EBM is a systematic and purposeful activity, each step of which should be promoted by clinical librarians working together with health care professionals [35]. Clinical librarians can play an important role in the EBM process, as their presence can serve to meet physicians' information needs and consequently improve the quality of care that is provided to patients [36]. Because the practice of EBM requires specialized knowledge and expertise, clinical librarians should be familiar with medical terminology, master the development of search strategies to find credible evidence, critically evaluate articles, have sufficient motivation to participate in medical teams, support accurate clinical decision-making, and be able to meet the clinical information needs of health care professionals in the shortest possible time [9–11, 20, 28, 34, 37]. Also, several studies emphasize the need not only to provide the best evidence to health care professionals and patients, but also to protect their privacy and confidentiality [3, 6, 7, 20].

### Communication excellence

Clinical librarians should strive to improve the quality of their communication with librarian colleagues and health care professionals by ensuring that their communication is constructive, useful, and respectful. At the same time, clinical librarians should welcome constructive criticism that they receive from colleagues and health care professionals, thereby bolstering the bidirectional nature of communication. Several previous studies have pointed to these issues and emphasize that clinical librarians should engage in appropriate and constructive communication with their colleagues, health care professionals, and patients [6, 20, 34]. This could be achieved in part by acquiring specialized knowledge about professional relationships and communication skills.

### Research excellence

The field of clinical librarianship must make use of validated research to improve the quality of its processes. To accomplish this, educational departments and research centers at universities should support EBM and clinical librarianship research to understand and meet the clinical information needs of health care professionals and to develop educational and research resources related to clinical librarianship and EBM with an emphasis on new concepts, theories, and local needs. Today's clinical librarians must base their activities and decisions on credible research and evidence. According to many studies, clinical librarians should not only present accurate and reliable evidence to physicians [7, 9, 10, 33–35, 37, 38], but they should also be involved in making evidence-based decisions by collecting, analyzing, and applying up-to-date research to their job processes. In addition, clinical librarians should position themselves to help physicians publish their research results.

### Professional status excellence

Professionals achieve success when their stakeholders benefit from their services. In other words, professional status is meaningless without contributions to others [39]. Clinical librarians should recognize that their primary beneficiaries are health care professionals and that their secondary beneficiaries are patients. Therefore, every effort to make accurate evidence available to health care professionals will ultimately lead to better care of patients. To achieve this goal, improving the quality of services, maintaining professional coherence, and establishing clinical librarians in health centers as well as striving to win the trust of health care professionals in the EBM-related capabilities of clinical librarians are of great importance. Thus, it is necessary to develop a philosophy, ideals, specialized knowledge, and professional standards for clinical librarians [6, 7, 20].

### Limitations

This study has some limitations. Although we attempted to identify experts to participate on the delphi panel, we may have failed to contact some key experts in clinical librarianship, which may have partially affected the results. Also, the lack of information related to the professional ethics of clinical librarians made it difficult to compile various sections of the questionnaire.

## CONCLUSION

Developing and promoting principles of professional ethics for clinical librarians can help the health care system balance the interests of all stakeholders, including clinical librarians, health care professionals, and patients. In the present study, we used a literature review and delphi panel to identify and approve five concepts and forty-six components of excellence in education, performance, communication, research, and professional status for clinical librarians. Our finding that the highest-rated concept was excellence in communication confirms the importance of communication in professional ethics. Also, the highest-rated component was mastery of search strategies in information resources and databases, suggesting that clinical librarians with more skills in finding information that physicians need are more successful in providing credible evidence to physicians and engender greater physician confidence in the profession of clinical librarianship.

Overall, identifying and striving to implement principles of professional ethics excellence for clinical librarians can enhance the professionalization of clinical librarians and result in better information services for physicians. Furthermore, incorporating these principles into the curriculum for health sciences library and information sciences students or into workshops for active clinical librarians can further formalize the profession and practice of EBM.

## Data Availability

The de-identified dataset and supporting files are available in Figshare at https://figshare.com/articles/Copy_of_round_1_4_xlsx/12091155.

## SUPPLEMENTAL FILES

**Appendix A:** Delphi first-round questionnaire (translation)

**Appendix B:** Delphi second-round questionnaire (translation)
