# Supplementary material for: Designing a model of professional ethics excellence for clinical librarians
Source: J Med Libr Assoc. 2020 Oct 1;108(4):574–83. doi: 10.5195/jmla.2020.893 (PMC7524624; doi:10.5195/jmla.2020.893)
Supplement: Supplementary file 1 — Appendix A: Delphi first-round questionnaire (translation) [file jmla-108-4-574-s01.pdf]

JMLA Journal of the Medical Library Association

SC
Supplemental content to
J Med Libr Assoc. Oct;108(4):dx.doi.org/10.5195/jmla.2020.893
www.jmla.mlanet.org
© Ashrafi-rizi, Kazempour, Sheikhshoaei, Ghazavi 2020

Designing a model of professional ethics excellence for clinical librarians

Hasan Ashrafi-rizi; Zahra Kazempour; Fatemeh Sheikhshoaei; Zahra Ghazavi, MSc

APPENDIX A

Delphi first-round questionnaire (translation)

In the Name of God

Dear colleague,

You are hereby informed that you are intended as an expert to provide constructive comments for research titled "Designing a Model of Professional Ethics Excellence for Clinical Librarians." The components of this model are derived from a literature review and interviews with some experts in the field. If you do not want to participate for some reason, let me know. Thanks in advance for your efforts. It is worth noting that the results of this questionnaire form the main part of this research that we are going to carry out at least in two rounds. Your personal information will remain confidential with the researcher. Therefore, your participation in answering the questions precisely will lead to the right results. Therefore, you are asked to give about fifteen to twenty minutes of your valuable time and complete this questionnaire in the first round of delphi to help us to do this research better. At the end of the main table, a table is also designed to present your suggestions so that you can present new suggested components. If you would like to access the results of this questionnaire or you need more information about the questionnaire and its analysis method, you can contact me by email (hassanashrafi@mng.mui.ac.ir or ashrafi.h89@gmail.com).

Thanks, Dr. Hassan Ashrafi-rizi, faculty member of Medical Library and Information Sciences Department, Isfahan University of Medical Sciences

Demographic features

Sex: Male ☐ Female ☐ Work experience (in years): _________

Level of education: MSc ☐ Doctoral (PhD) student ☐ PhD ☐

Academic rank: Instructor☐ Assistant professor ☐ Associate professor ☐ Full professor ☐ Other ☐

Field of study: Medical library and information sciences ☐ Library and information sciences ☐ Information sciences and knowledge studies ☐ Other ☐

Your university name: _______________ Your school name: _______________

Please indicate your acceptance rate of the following components for "Excellence of Professional Ethics for Clinical Librarians"

SC
Supplemental content to
J Med Libr Assoc. Oct;108(4):dx.doi.org/10.5195/jmla.2020.893
www.jmla.mlanet.org
© Ashrafi-rizi, Kazempour, Sheikhshoaei, Ghazavi 2020

Concept	Component	Low importance →									Very important
		1	2	3	4	5	6	7	8	9	10
Education excellence	1. Converts health care professionals into practitioners of evidence-based medicine (EBM)										
	2. Continues to educate health care professionals about clinical information literacy										
	3. Continues to educate residents and interns about clinical information literacy										
	4. Updates one's specialized knowledge and that of other librarians by participating in continuing education										
	5. Engages in practical in-service training in clinical librarianship										
	6. Recommends and updates medical librarianship curriculums with a focus on clinical librarianship										
	7. Trains a new generation of clinical librarians to support EBM										
	8. Takes advantage of the knowledge and expertise of leaders in clinical librarianship										
Performance excellence	9. Demonstrates relative command of terms and concepts used by health care professionals										
	10. Masters search strategies in information resources and databases										
	11. Masters clinical question formation (e.g., problem, intervention, comparison, outcome [PICO])										
	12. Demonstrates sufficient knowledge and mastery of evidence-based information sources										

SC
Supplemental content to
J Med Libr Assoc. Oct;108(4):dx.doi.org/10.5195/jmla.2020.893
www.jmla.mlanet.org
© Ashrafi-rizi, Kazempour, Sheikhshoaei, Ghazavi 2020

Concept	Component	Low importance →									Very important
		1	2	3	4	5	6	7	8	9	10
	13. Pays attention to the clinical information needs of health care professionals										
	14. Provides reliable and up-to-date information (i.e., best evidence) for health care professionals										
	15. Considers patient values in the EBM process										
	16. Quickly and accurately responds to the clinical questions of health care professionals										
	17. Supports accurate clinical decisions and records experiences										
	18. Attends clinical rounding										
	19. Maintains patient privacy										
	20. Believes in the role and influence of clinical information services on the level of care received by patients										
	21. Has a timely and effective presence in the clinical setting										
Communication excellence	22. Appropriately and respectfully communicates with health care professionals and patients										
	23. Enjoys good self-esteem when interacting with health care professionals										
	24. Shows confidence in communicating with health care teams										
	25. Collaborates with health care teams to facilitate the EBM process										
	26. Utilizes one's scientific ability and talent and that of other librarians to provide appropriate services to health care professionals										

JMLA Journal of the Medical Library Association

Supplemental content to
J Med Libr Assoc. Oct;108(4):dx.doi.org/10.5195/jmla.2020.893
www.jmla.mlanet.org
© Ashrafi-rizi, Kazempour, Sheikhshoaei, Ghazavi 2020

Concept	Component	Low importance → Very important									
		1	2	3	4	5	6	7	8	9	10
	27. Actively interacts with and shows respect to other librarians										
	28. Accepts constructive and wise feedback from other librarians and health care professionals										
	29. Respects the job performance of other librarians										
	30. Has insight into the information behavior and performance of health care professionals										
	31. Avoids inappropriate jokes when performing job activities										
	32. Has a professional physical appearance in the workplace										
	33. Effectively communicates with senior executives of the organization to support and enhance EBM and clinical librarianship										
	34. Gains the necessary communication skills for interacting with others										
	35. Is available even during non-office hours when necessary										
Research excellence	36. Supports research related to clinical librarianship and EBM										
	37. Supports rationale for using best evidence in clinical decision-making										
	38. Identifies clinical information needs of health care professionals based on scientific research										
	39. Develops scientific and teaching resources related to clinical librarianship and EBM with an emphasis on new concepts, theories, and local needs										

SC
Supplemental content to
J Med Libr Assoc. Oct;108(4):dx.doi.org/10.5195/jmla.2020.893
www.jmla.mlanet.org
© Ashrafi-rizi, Kazempour, Sheikhshoaei, Ghazavi 2020

Concept	Component	Low importance ⟶ Very important									
		1	2	3	4	5	6	7	8	9	10
	40. Develops clinical librarian programs and services based on valid research findings										
Professional status excellence	41. Believes in the existential philosophy of clinical librarianship to effectively enhance clinical information services provided to health care professionals										
	42. Believes in evidence-based clinical librarianship and its formalization										
	43. Gains the trust of health care professionals in the capabilities of clinical librarians in the context of EBM										
	44. Strengthens and expands the independent and effective identity of clinical librarians among the general public and health care professionals										
	45. Believes in professional cohesion and moving toward common interests										
	46. Strives to maximize the usefulness of clinical information services										
	47. Strives to promote the status of clinical librarians at national and international levels										
	48. Upgrades the level of professional integration of clinical librarianship										

JMLA Journal of the Medical Library Association

SC
Supplemental content to
J Med Libr Assoc. Oct;108(4):dx.doi.org/10.5195/jmla.2020.893
www.jmla.mlanet.org
© Ashrafi-rizi, Kazempour, Sheikhshoaei, Ghazavi 2020

Please suggest the other concepts and components and indicate your acceptance rate with them

**Concept**	**Component**	**Low importance** →									**Very important**
		**1**	**2**	**3**	**4**	**5**	**6**	**7**	**8**	**9**	**10**
