# Supplementary material for: Designing a model of professional ethics excellence for clinical librarians
Source: J Med Libr Assoc. 2020 Oct 1;108(4):574–83. doi: 10.5195/jmla.2020.893 (PMC7524624; doi:10.5195/jmla.2020.893)
Supplement: Supplementary file 2 — Appendix B: Delphi second-round questionnaire (translation) [file jmla-108-4-574-s02.pdf]

JMLA Journal of the Medical Library Association

SC
Supplemental content to
J Med Libr Assoc. Oct;108(4):dx.doi.org/10.5195/jmla.2020.893
www.jmla.mlanet.org
© Ashrafi-rizi, Kazempour, Sheikhshoaei, Ghazavi 2020

Designing a model of professional ethics excellence for clinical librarians

Hasan Ashrafi-rizi; Zahra Kazempour; Fatemeh Sheikhshoaei; Zahra Ghazavi, MSc

APPENDIX B

Delphi second-round questionnaire (translation)

In the Name of God

Dear colleague,

Thank you for your valuable comments in the first phase of the delphi panel. This questionnaire is set based on delphi members' opinions on "Designing a Model of Professional Ethics Excellence for Clinical Librarians" in the first phase. In the second phase, we would like to use your valuable comments again. It is hoped that the results of this study can add to the richness of the professional ethics of the clinical librarians. So please complete the questionnaires carefully. Thank you very much for giving your valuable time in this research. If you have any questions or concerns, contact me by email (hassanashrafi@mng.mui.ac.ir or ashrafi.h89@gmail.com).

Thanks, Dr. Hassan Ashrafi Rizi, faculty member of Medical Library and Information Sciences Department, Isfahan University of Medical Sciences

Changes made to the questionnaire after the first delphi phase

1. In the first phase, some components got a mean lower than 7, like "Availability even in non-office hours when necessary" with 5.60 mean, "Recommending and collaborating in updating medical librarianship curriculums with a focus on clinical librarianship education" with 6.72 mean, and "Training a new generation of clinical librarians to support EBM" with 7.00 mean. If they do not get a mean higher than 7 in this phase, they will be eliminated from the "Model of Professional Ethics Excellence for Clinical Librarians."
2. We corrected the components in terms of problem in concept or writing (7 components).
3. Another key concept was introduced by a panel member along with 2 components, with the title of "Complying with the rules governing clinical and treatment settings."
4. A component was added to concept of "performance excellence."
5. The mean of every component in first phase was added to this questionnaire for informing the delphi panel members.

Second phase of delphi

1. In this phase, it is necessary you state your final view about the components in the following tables.
2. In second table, there are the components suggested by delphi panel members in first phase; please state your valuable views about them.

Please indicate your acceptance rate of the following components for "Excellence of Professional Ethics for Clinical Librarians"

JMLA Journal of the Medical Library Association

SC
Supplemental content to
J Med Libr Assoc. Oct;108(4):dx.doi.org/10.5195/jmla.2020.893
www.jmla.mlanet.org
© Ashrafi-rizi, Kazempour, Sheikhshoaei, Ghazavi 2020

Concept	Component	Mean of first phase	Low importance → Very important									
			1	2	3	4	5	6	7	8	9	10
Education excellence	1. Converts health care professionals into practitioners of evidence-based medicine (EBM)	7.60										
	2. Continues to educate health care professionals about clinical information literacy	8.40										
	3. Continues to educate residents and interns about clinical information literacy	8.12										
	4. Updates one's specialized knowledge and that of other librarians by participating in continued education	9.09										
	5. Engages in practical in-service training in clinical librarianship	8.60										
	6. Recommends and updates medical librarianship curriculums with a focus on clinical librarianship	6.72										
	7. Trains a new generation of clinical librarians to support EBM	7.00										
	8. Takes advantage of the knowledge and expertise of leaders in clinical librarianship	7.45										
Performance excellence	9. Demonstrates relative command of the terms and concepts used by health care professionals	8.27										

JMLA Journal of the Medical Library Association

SC
Supplemental content to
J Med Libr Assoc. Oct;108(4):dx.doi.org/10.5195/jmla.2020.893
www.jmla.mlanet.org
© Ashrafi-rizi, Kazempour, Sheikhshoaei, Ghazavi 2020

Concept	Component	Mean of first phase	Low importance → Very important									
			1	2	3	4	5	6	7	8	9	10
	10. Masters search strategies in information resources and databases	8.72										
	11. Masters clinical question formation (e.g., problem, intervention, comparison, outcome [PICO])	8.40										
	12. Demonstrates sufficient knowledge and mastery of evidence-based information sources	8.27										
	13. Pays attention to clinical information needs of health care professionals	8.27										
	14. Provides reliable and up-to-date information (i.e., best evidence) for health care professionals	8.20										
	15. Considers patient values in the EBM process	7.80										
	16. Quickly and accurately responds to the clinical questions of health care professionals	8.18										
	17. Supports accurate clinical decisions and records experiences	7.63										
	18. Attends clinical rounding	8.00										
	19. Maintains patient privacy	8.63										
	20. Believes in the role and influence of clinical information services on the level of care received by patients	9.18										

JMLA Journal of the Medical Library Association

Supplemental content to
J Med Libr Assoc. Oct;108(4):dx.doi.org/10.5195/jmla.2020.893
www.jmla.mlanet.org
© Ashrafi-rizi, Kazempour, Sheikhshoaei, Ghazavi 2020

Concept	Component	Mean of first phase	Low importance → Very important									
			1	2	3	4	5	6	7	8	9	10
	21. Has a timely and effective presence in the clinical setting	8.00										
Communication excellence	22. Appropriately and respectfully communicates with health care professionals and patients	8.36										
	23. Enjoys good self-esteem when interacting with health care professionals	8.36										
	24. Shows confidence in communicating with health care teams	8.45										
	25. Collaborates with health care teams to facilitate the EBM process	9.00										
	26. Utilizes one's scientific ability and talent and that of other librarians to provide appropriate services to health care professionals	7.90										
	27. Actively interacts with and shows respect to other librarians	7.81										
	28. Accepts constructive and wise feedback from other librarians and health care professionals	8.45										
	29. Respects the job performance of other librarians	7.90										
	30. Has insight into the information behavior and performance of health care professionals	7.54										

JMLA Journal of the Medical Library Association

SC
Supplemental content to
J Med Libr Assoc. Oct;108(4):dx.doi.org/10.5195/jmla.2020.893
www.jmla.mlanet.org
© Ashrafi-rizi, Kazempour, Sheikhshoaei, Ghazavi 2020

Concept	Component	Mean of first phase	Low importance → Very important									
			1	2	3	4	5	6	7	8	9	10
	31. Avoids inappropriate jokes when performing job activities	7.27										
	32. Has a professional physical appearance in the workplace	7.90										
	33. Effectively communicates with senior executives of the organization to support and enhance EBM and clinical librarianship	8.18										
	34. Gains the necessary communication skills for interacting with others	8.11										
	35. Is available even during non-office hours when necessary	5.60										
Research excellence	36. Supports research related to clinical librarianship and EBM	8.27										
	37. Supports rationale for using best evidence in clinical decision-making	8.63										
	38. Identifies clinical information needs of health care professionals based on scientific research	8.27										
	39. Develops scientific and teaching resources related to clinical librarianship and EBM with an emphasis on new concepts, theories, and local needs	7.18										
	40. Develops clinical librarian programs and services based on valid research findings	8.36										

JMLA Journal of the Medical Library Association

SC
Supplemental content to
J Med Libr Assoc. Oct;108(4):dx.doi.org/10.5195/jmla.2020.893
www.jmla.mlanet.org
© Ashrafi-rizi, Kazempour, Sheikhshoaei, Ghazavi 2020

Concept	Component	Mean of first phase	Low importance ⟶ Very important									
			1	2	3	4	5	6	7	8	9	10
Professional status excellence	41. Believes in the existential philosophy of clinical librarianship to effectively enhance clinical information services provided to health care professionals	8.63										
	42. Believes in evidence-based clinical librarianship and its formalization	8.45										
	43. Gains the trust of health care professionals in the capabilities of clinical librarians in the context of EBM	7.63										
	44. Strengthens and expands the independent and effective identity of clinical librarians among the general public and health care professionals	8.00										
	45. Believes in professional cohesion and moving toward common interests	7.90										
	46. Strives to maximize the usefulness of clinical information services	8.27										
	47. Strives to promote the status of clinical librarians at national and international levels	8.27										
	48. Upgrades the level of professional integration in clinical librarianship	8.00										

JMLA Journal of the Medical Library Association

Supplemental content to
J Med Libr Assoc. Oct;108(4):dx.doi.org/10.5195/jmla.2020.893
www.jmla.mlanet.org
© Ashrafi-rizi, Kazempour, Sheikhshoaei, Ghazavi 2020

Please indicate your acceptance rate of the components suggested by colleagues in the first phase.

		**Low importance**			**⟶**						**Very important**
**Concept**	**Component**	**1**	**2**	**3**	**4**	**5**	**6**	**7**	**8**	**9**	**10**
Performance excellence	Feedback of clinical librarian's presence and its impact in treatment process										
Strives to enforce the rules governing the clinical setting and treatment	Works in line with the standards of work environment (e.g. hospital, clinic, etc.)										
	Pays attention to the guidelines governing the treatment environment										
